# Oral sarcoidosis preceding sudden cardiac arrest: a case report

**DOI:** 10.1093/ehjcr/ytae557

**Published:** 2024-10-23

**Authors:** William Swain, Matteo Castrichini, Konstantinos Siontis, Fadi Hasan, Courtney Arment

**Affiliations:** Department of Internal Medicine, Mayo Clinic, 200 1st St SW, Rochester, MN 55905, USA; Department of Internal Medicine, Mayo Clinic, 200 1st St SW, Rochester, MN 55905, USA; Department of Cardiovascular Medicine, Mayo Clinic, 200 1st St SW, Rochester, MN 55905, USA; Department of Cardiovascular Medicine, Mayo Clinic, 200 1st St SW, Rochester, MN 55905, USA; Sculpt Center Implants & Periodontics, 8201 Greensboro Dr, Suite 702, McLean, VA 22102, USA; Department of Rheumatology, Mayo Clinic, 200 1st St SW, Rochester, MN 55905, USA

**Keywords:** Cardiac sarcoidosis, Case report, Oral sarcoidosis, Sudden cardiac arrest

## Abstract

**Background:**

Sarcoidosis is a disease characterized by non-caseating granulomas and may affect any organ system. Cardiac involvement may lead to conduction abnormalities, heart failure, or malignant ventricular arrhythmias. As sarcoidosis may present with heterogeneous manifestations, a detailed past medical history may provide clues that help guide further workup. We present a rare case of a patient with undiagnosed oral sarcoidosis who subsequently experienced cardiac arrest from cardiac involvement.

**Case summary:**

A 43-year-old male with a history of palpitations and periodontitis consistent with oral sarcoidosis presents after experiencing sudden cardiac arrest. He was subsequently diagnosed with cardiac and pulmonary sarcoidosis. With contemporary management (both immunosuppression and antiarrhythmics), he has not experienced any recurrent arrhythmias.

**Discussion:**

In the setting of cardiac arrest and non-ischaemic cardiomyopathy, a careful clinical history and targeted cardiac testing may help clinicians determine when to consider cardiac sarcoidosis as a diagnosis. While oral sarcoidosis is a very rare condition, this case highlights how infrequent manifestations of sarcoidosis may be encountered in the clinical setting.

**Learning points**

- To illustrate the importance of a careful clinical history in the workup for cardiac sarcoidosis.
- To detail findings that may lend to consideration of cardiac sarcoidosis in the setting of non-ischaemic cardiomyopathy and cardiac arrest.
- To demonstrate how to screen for cardiac involvement in patients with known systemic sarcoidosis through clinical history, electrocardiogram, and cardiac imaging.

## Introduction

Sarcoidosis is characterized by granulomatous inflammation of non-infectious aetiology. It can affect virtually any organ system, leading to a variety of clinical presentations.^1^ We present a rare case of oral sarcoidosis that went undiagnosed before cardiac involvement was discovered, highlighting the importance of a careful clinical history (Summary figure).

## Summary figure

**Table:** 

Date	Event
−10 years	The patient has longstanding palpitations but had never discussed these with a physician.
−2 years	Patient was diagnosed with periodontitis. He underwent gum-grafting surgery that failed due to infection.
−4 months	After his infection healed, the patient had re-do bone and gum grafting for his periodontitis. A biopsy of the gingiva showed granulomatous inflammation. The meaning of this was uncertain at the time.
Day 0	Patient experienced cardiac arrest due to pulseless ventricular tachycardia and underwent bystander cardio-pulmonary resuscitation. He is admitted to the hospital where coronary angiography showed no significant stenosis.
Day 1	Transthoracic echocardiography demonstrated ejection fraction of 35% with mild global hypokinesis.
Days 3–7	Cardiac MRI showed epicardial late gadolinium enhancement in a non-coronary distribution. Incidentally, enhancing splenic nodules were seen. Chest CT showed micronodularity in the right upper lung in a perilymphatic distribution. An implantable cardioverter-defibrillator was placed prior to discharge, and he was started on prednisone 40 mg with taper, and loaded on oral amiodarone.
Week 5	Outpatient right upper lobe wedge resection is done, which showed non-caseating granulomas of non-infectious aetiology consistent with sarcoidosis. He is started on methotrexate 15 mg weekly and mexiletine 150 mg daily.
Month 3	Amiodarone was tapered off, mexiletine continued.
Month 4	Prednisone was weaned off, methotrexate continued.
18 months	The patient remains on methotrexate and mexiletine. He has not experienced any recurrent ventricular arrhythmias.

## Case presentation

**Figure 1:**
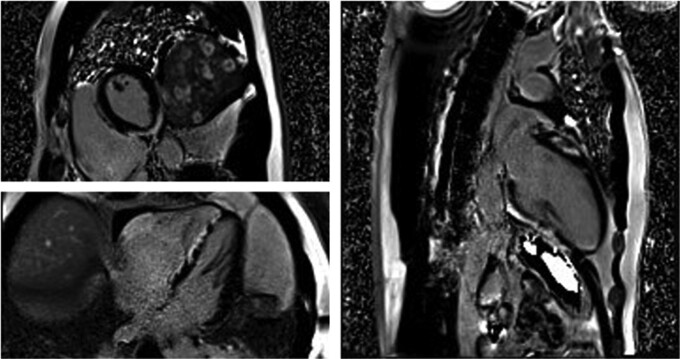
MRI phase contrast inversion recovery images showing multifocal late gadolinium enhancement in the septum and subepicardial and mid-myocardial regions of the basal inferior and anterior walls, consistent with cardiac sarcoidosis. The basal inferior wall also has associated oedema on T2 imaging suggesting ongoing inflammation.

A 43-year-old male presented after experiencing witnessed cardiac arrest and bystander cardio-pulmonary resuscitation. When emergency medical services arrived, he was in ventricular tachycardia and was successfully cardioverted after five defibrillator shocks (the last one being dual-sequential at 200 J). Upon arrival to the hospital, he was unresponsive to commands. Auscultation revealed an elevated heart rate and clear breath sounds. He was loaded with amiodarone, underwent general anaesthesia, and was started on norepinephrine and dobutamine. Coronary angiography revealed no significant coronary artery stenoses. Echocardiogram demonstrated left ventricular ejection fraction (LVEF) 35% with diffuse hypokinesis and akinesis of the basal-inferior wall. Cardiac magnetic resonance imaging (CMR) showed areas of extensive late gadolinium enhancement (LGE) in the septum and the subepicardial and mid-myocardial inferior wall at the base and mid-ventricle (*Figure 1*). Innumerable enhancing splenic lesions were also seen.

**Figure 2:**
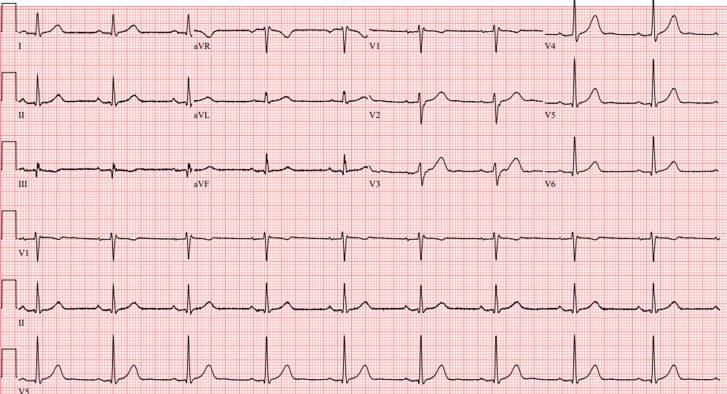
Resting electrocardiogram obtained after hospitalization shows borderline first-degree atrioventricular block (PR interval = 204 ms) and non-specific intraventricular conduction delay.

The patient had no previous past medical history. He did endorse palpitations for the past 10 years but denied a history of cough, shortness of breath, swelling, or lymphadenopathy. He also denied a family history of premature coronary artery disease, non-ischaemic cardiomyopathy, and sudden cardiac death. He was a non-smoker, denied heavy alcohol use, and exercised regularly. After stabilization, he had normal serum creatinine, calcium, vitamin D, troponin, liver function, and angiotensin-converting enzyme levels. His resting electrocardiogram (ECG) demonstrated borderline first-degree atrioventricular block with non-specific intraventricular conduction delay (*Figure 2*). Given the patient’s relatively young age with splenic findings, a diagnosis of cardiac sarcoidosis was considered. Chest computed tomography (CT) showed micronodularity in the right upper and mid-lung in a perilymphatic distribution consistent with pulmonary sarcoidosis. Before discharge, he was started on amiodarone 400 mg daily, metoprolol succinate 50 mg daily, prednisone 40 mg daily, and an implantable cardioverter-defibrillator (ICD) was placed. Further pharmacologic therapy for heart failure was deferred given recovery of LVEF to 55% prior to discharge.

At post-discharge follow-up 1 week later, his LVEF was 65%, suggesting that his previously low LVEF was due to cardiac arrest and not another underlying cause. Genetic testing for non-ischaemic cardiomyopathies was negative. The right upper lung was biopsied via wedge resection, showing fibrosis with granulomata and numerous multinucleated giant cells with negative methenamine-silver and Fite stains. A diagnosis of systemic sarcoidosis with probable cardiac involvement was made according to the 2014 Heart Rhythm Society criteria.^2^ Methotrexate 15 mg weekly and mexiletine 150 mg three times daily were started. Cardiac positron emission tomography (PET) CT performed 1 month later showed no 18-Fludeoxyglucose (18-FDG) uptake in the myocardium, although the patient was on immunosuppression (methotrexate and prednisone) at this time. Amiodarone was discontinued at 3 months post-arrest, and he remained on mexiletine. During a follow-up of 18 months, the patient did not have recurrent ventricular arrhythmias or further sequelae of heart failure.

**Figure 3:**
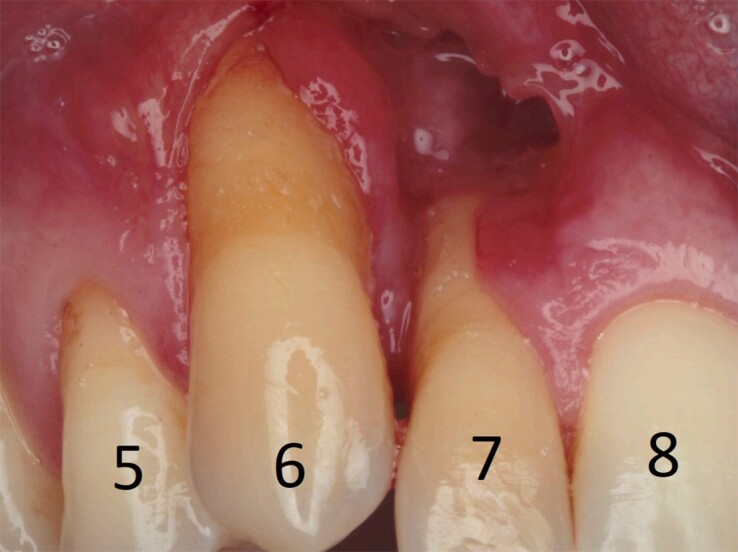
Severe periodontitis from oral sarcoidosis, with severe gum recession at teeth #6 and #7. The missing gingival tissue above tooth #7 was from a prior gum graft infection that required debridement.

**Figure 4:**
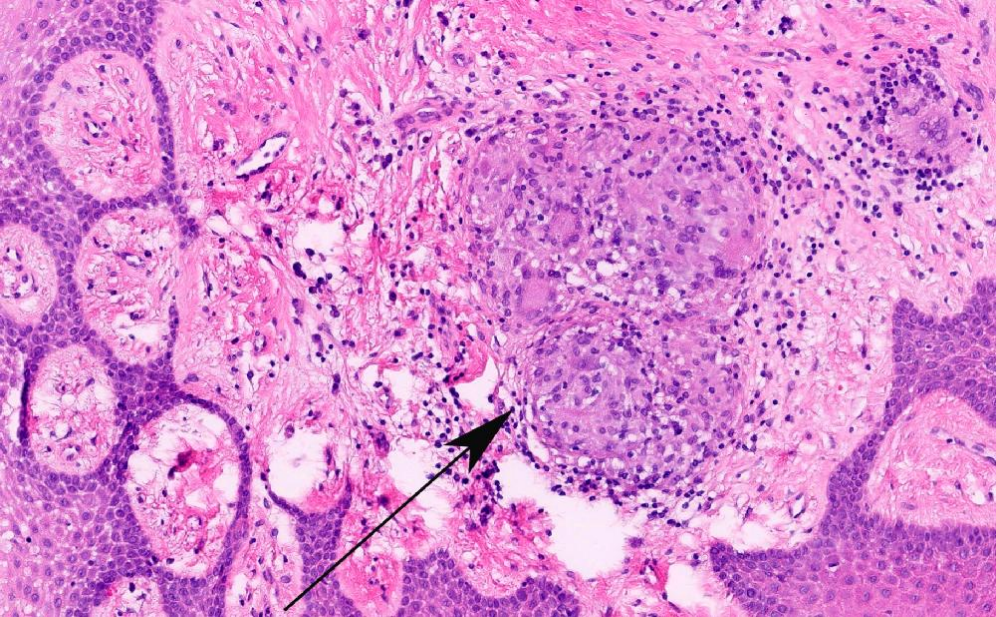
Haematoxylin and eosin stain of gingival mucosa magnified at 200× showing submucosal evidence of chronic inflammation (black arrow). Within the milieu of chronic inflammation are non-necrotizing granulomas. No micro-organisms were seen on subsequent periodic acid-Schiff, acid-fast bacilli, and methenamine-silver stains.

Unbeknownst to the physicians who treated this patient in the post-arrest period, the patient had a significant dental history. During the two years prior to arrest, he had experienced severe periodontitis and underwent numerous dental procedures complicated by poor healing. Just 4 months prior to arrest, a biopsy of the gingival mucosa was taken during a bone and gum grafting procedure (*Figure 3*). This showed granulomas composed of histiocytes and giant cells (*Figure 4*). The patient was aware that he had ‘granulomatous inflammation’ of his gums but had not discussed the pathology report with his physician yet.

## Discussion

Oral sarcoidosis is very rare. As of 2014, there were only 70 reported cases in the literature, although this is likely under-reported.^3^ It can present as nodules or ulcers in the buccal mucosa, gingiva, soft and hard palates, tongue, lips, jaw bones, and glands.^4^ Sometimes it may be the initial manifestation of systemic sarcoidosis.^5^ Most commonly, oral granulomas are due to a foreign-body reaction to dental material, although, as illustrated by our case, these can be indicative of an underlying systemic disease.^6,7^ Clinicians should be aware of the less common manifestations of sarcoidosis to prevent missed diagnoses and potential downstream adverse consequences. This patient had a first-degree atrioventricular block on ECG and may have benefited from further workup of his palpitations. An earlier diagnosis of cardiac sarcoidosis might have prevented his cardiac arrest through sudden cardiac death risk stratification based on LVEF and the amount of LGE on CMR present. Implantable cardioverter-defibrillator implementation for primary prevention in cardiac sarcoidosis should be considered if significant LGE is present after treatment of acute inflammation, and electrophysiologic testing may be considered when LVEF is 35%–50% and minor LGE is present.^8^

In the patient presenting with resuscitated cardiac arrest and normal coronary arteries, the differential diagnosis is guided by clinical history, family history, and multi-modality imaging. Suspicion for cardiac sarcoidosis may increase in the setting of unexplained cough, dyspnoea, pulmonary infiltrates, hilar lymphadenopathy, skin lesions, and ECG abnormalities but none of these findings are specific.^9^ Unexplained laboratory, imaging findings, or histological evidence of granulomatous inflammation elsewhere in the body may also lend towards consideration of cardiac sarcoidosis.

While cardiac involvement may be present in up to a quarter of those with systemic sarcoidosis, the presence of clinically relevant disease is ∼5%.^2,10^ Cardiac sarcoidosis is typically diagnosed based on histologic confirmation of extra-cardiac tissue with evidence of cardiac involvement on CMR or PET imaging.^2^ Endomyocardial biopsy is specific, but suffers from low sensitivity (20%–30%) even when performed with electrogram mapping given the patchy nature of the disease.^11^

In the setting of biopsy-proven extra-cardiac sarcoidosis, clinicians should inquire about unexplained cardiac symptoms (syncope, presyncope, or palpitations). A baseline ECG should be obtained, and an echocardiogram should be considered.^2^ Ambulatory Holter monitoring to screen for arrhythmias and ectopy may be useful, especially in patients with paroxysmal symptoms.

Electrocardiogram features suggestive of cardiac sarcoidosis include atrioventricular conduction disease, bundle branch blocks, and ventricular ectopy. Echocardiogram features include regional wall motion abnormalities, wall aneurysm or thinning, and left or right ventricular systolic dysfunction. If any abnormalities are uncovered, then CMR and/or 18-FDG PET-CT should be considered. On CMR, patchy LGE in a non-coronary distribution that spares the endocardium (commonly affecting the basal septum and right ventricular insertion points) is suggestive but non-specific for cardiac sarcoidosis.^12,13^ 18-FDG PET-CT may demonstrate focal uptake with or without a background of diffuse mild uptake. Serial PET imaging allows for assessing therapy response, and is preferred in patients with ICDs due to less imaging artefact.^13^ Additionally, whole-body PET allows for visualization of extra-cardiac tissues which may be affected.^12^ PET imaging, however, can only identify active inflammation and may be less useful in immunosuppressed patients as illustrated in our case. While steroids and other immunosuppressants are often used to treat ventricular arrhythmias, there is mixed evidence on their efficacy.^2^ After surviving a cardiac arrest, antiarrhythmic medications are generally indicated and catheter ablation may be indicated depending on the clinical scenario.^14^

This case highlights the broad spectrum of clinical manifestations that may be encountered in sarcoidosis, and how a thorough medical history and even dental history may guide clinicians in the setting of suspected sarcoidosis.

## Lead author biography


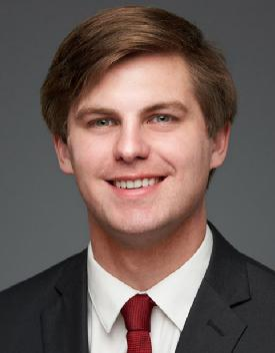


William Swain is a third-year internal medicine resident at the Mayo Clinic in Rochester, Minnesota, USA. He obtained his undergraduate degree in biomedical engineering from the University of Michigan, and his medical degree from the University of Wisconsin School of Medicine and Public Health. He has a keen interest in cardiology and electrophysiology.

## Data Availability

No new data were created or analysed in support of this research.
